# Image Super-Resolution via Dual-Level Recurrent Residual Networks

**DOI:** 10.3390/s22083058

**Published:** 2022-04-15

**Authors:** Congming Tan, Liejun Wang, Shuli Cheng

**Affiliations:** 1 College of Information Science and Engineering, Xinjiang University, Urumqi 830046, China; smartan1997@stu.xju.edu.cn (C.T.); cslxju@xju.edu.cn (S.C.); 2 College of Mathematics and System Science, Xinjiang University, Urumqi 830046, China

**Keywords:** super-resolution, dual-level, satisfactory vision

## Abstract

Recently, the feedforward architecture of a super-resolution network based on deep learning was proposed to learn the representation of a low-resolution (LR) input and the non-linear mapping from these inputs to a high-resolution (HR) output, but this method cannot completely solve the interdependence between LR and HR images. In this paper, we retain the feedforward architecture and introduce residuals to a dual-level; therefore, we propose the dual-level recurrent residual network (DLRRN) to generate an HR image with rich details and satisfactory vision. Compared with feedforward networks that operate at a fixed spatial resolution, the dual-level recurrent residual block (DLRRB) in DLRRN utilizes both LR and HR space information. The circular signals in DLRRB enhance spatial details by the mutual guidance between two directions (LR to HR and HR to LR). Specifically, the LR information of the current layer is generated by the HR and LR information of the previous layer. Then, the HR information of the previous layer and LR information of the current layer jointly generate the HR information of the current layer, and so on. The proposed DLRRN has a strong ability for early reconstruction and can gradually restore the final high-resolution image. An extensive quantitative and qualitative evaluation of the benchmark dataset was carried out, and the experimental results proved that our network achieved good results in terms of network parameters, visual effects and objective performance metrics.

## 1. Introduction

Image super-resolution (SR), reconstructing HR from the corresponding LR image, is an important image processing technique in computer vision. It has applications in all aspects of the real world, such as medical imaging [1], surveillance and security [2] and satellite imaging [3].

The SR task has the inherent ill-posed problem that multiple different HR images can be recovered from a single LR image. To solve this issue, researchers have proposed a number of methods for SR reconstruction, which we can divide into two categories according to the process of reconstruction: traditional-based methods (such as interpolation-based methods [4] or reconstruction-based methods [5]) and learning-based methods (DL). At present, the typical method is to learn the non-linear mapping of LR-HR through neural networks [2,6,7,8] to construct HR images. These networks calculate a series of feature maps from LR images; the resolution is then increased by one or more upsampling layers to construct the final HR images. Compared with these pure feedforward methods, it is believed that using a feedback connection to simply guide the tasks can produce results that are more suitable for the human visual system, i.e., visually satisfactory results [9].

Dong et al. [10] first used the CNN model for the SR task and the proposed SRCNN, which predicts the non-linear mappings of LR-HR via a fully connected layers network. Its reconstruction results are significantly better than traditional methods. The advantage of the deep learning method comes from two key factors. Firstly, increasing the depth of the CNN model to learn more complex mappings from LR to HR and to improve SR performance. Secondly, adding residual connections to the network (globally [7], locally [11] or jointly [8]) can effectively alleviate the problem of gradient vanishing and exploding caused by deepening the network only by stacking more layers.

Although these methods based on deep learning can achieve superior results, there are also some shortcomings. The main problem is that the deeper the network, the more parameters are required, and the more storage resources are taken up. A recursive structure is usually adopted to reduce network parameters. These networks with recursive structures work at a single spatial resolution (e.g., DRCN [7] and DRRN [8]). Similar to most CNN-based approaches, these networks transmit information in a feedforward manner. 

**Figure 1:**
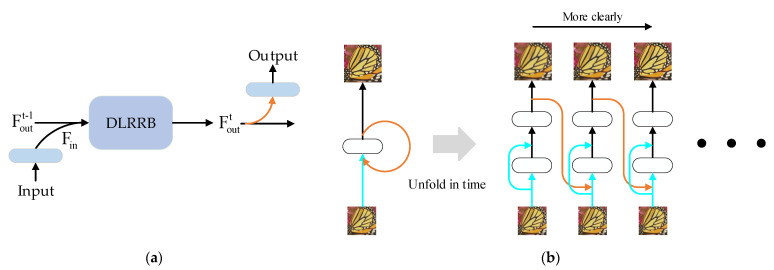
The illustration of the feedback mechanism in the proposed network. (**a**) Feedback is carried out through the hidden information in DLRRB in one iteration. (**b**) The principles of our feedback scheme, which gradually reconstruct a clearer image; orange arrows represent the feedback connection.

In this paper, we add an additional level to the residual branch in the classical feedforward network structure, so that our model becomes a dual-level network that operates in different resolution spaces. Specifically, the HR-level (HRL) information is used to refine LR-level (LRL) information through feedback connections, while it uses LRL to enrich HRL information through feedforward connections, and finally, obtains SR with rich details and is visually satisfied. The DLRRB is composed of multiple groups of cross-level feature fusion blocks of HRL (CLFFB_S) and cross-level feature fusion blocks of LRL (CLFFB_L) with dense connections. We use the output of CLFFB_S (that is, the hidden information of the DLRRB as shown in Figure 1a) as the feedback information in our network. The hidden information ($F\_lr_{out}^{t}$ and $F\_sr_{out}^{t}$) in the DLRRB of each iteration was used to modulate the input of the next iteration and output $F\_sr_{out}^{t}$. To provide our network with an early reconstruction ability and obtain clearer SR images, as in work [12], we input the LR images into each iteration and formed loss functions between the output SR and HR in each iteration. The principle of the feedback scheme in our network is that the HRL information in the feedback information flow can refine the LR image features, and the refined LR image features can guide the network to gradually construct better SR images. Our network ranks successive iterations of target HR images from easy to hard according to the difficulty of the LR image recovery. Such a learning process allows our network DLRRN to handle a more complex degradation, while the experimental results also prove that our network can deal accurately with complex degradation models.

The DLRRN proposed in this paper is different from DSRN [13] in the following three points: Firstly, this paper performs mutual correction through the feature map of the image, while the image directly processed by DSRN will increase the memory consumption of the network. Secondly, the DLRRN proposed in this paper outputs SR images at each iteration, which provides the network with the ability of early reconstruction and can deal with more complex degradation. Thirdly, this paper uses the output of the last iteration as the final output image, while the final output of DSRN is the LR image, and then the final output is obtained by upsampling. In general, the DLRRN and DSRN are very different in terms of performance and network structure.

In summary, our main contributions are as follows:

- This paper proposes a single image super-resolution network via dual-level recurrent residuals (DLRRN), which use both feedforward and feedback connections to generate HR images with rich details. This recursive structure with feedback connections has a small number of parameters, while providing a powerful early reconstruction capability.
- Inspired by [14], in this paper, a cross-layer feature fusion block (CLFFB) for the SR task is designed as the core part of DLRRB, which can enhance information by effectively processing cross-layer information flow.
- Since the self-attention module [15] can describe the spatial correlation of any two positions in an image, in this paper, we use it to propose the self-attention feature extraction block (SAFEB). SAFEB models the local features by contextual relevance; it cooperates with the applied MS-SSIM [16] to improve the reconstruction performance and produce better visual effects.

The remainder of this paper is arranged as follows: The second section mainly introduces some classic super-resolution algorithms based on deep learning and attempts to apply feedback connections to super-resolution, as carried out in recent years. The third section is the details of our network. The fourth section is about the implementation details of our experiment and the analysis of the results. The fifth section is the summary of this paper and some defects of the algorithm. 

## 2. Related Work

### 2.1. Deep-Learning-Based Image Super-Resolution

Due to the powerful learning ability of deep learning, many scholars have introduced it into computer vision tasks (including SR), and the results have shown its excellent performance. Dong et al. [10] proposed the first CNN-based SR method, namely SRCNN, which introduced three fully connected layers to SR tasks to learn the complex mapping from LR to HR, and SRCNN was trained via end-to-end methods. Theoretically, the CNN-based SR network reconstruction process consists of three stages: feature extraction, non-linear mapping and image reconstruction. The VDSR proposed by Kim et al. [6] learns the LR to HR representation by stacking 20 convolutional layers. In [8], a skip connection and adjustable gradient are adopted to overcome gradient vanishing and exploding, which may be caused when the network becomes deeper. However, the deeper the model, the more parameters it needs, which is not conducive to practical applications. It has become a research hotspot for reducing network parameters without sacrificing network performance, the DRCN [7] loops the same recursive layer 16 times, which can effectively reduce parameters without reducing network performance. In addition, skip connections and recursive supervision are used in DRCN to alleviate training difficulties. A variety of different skip connections are used in SR tasks to improve reconstruction performance. The residual skip connections in [17] were applied to SRResNet [18] and EDSR [19]. SRDenseNet [20] applies the dense skip connections in [21]. Zhang et al. [22] proposed RDN using local/global residuals and dense skip connections. These network structures can use or combine hierarchical features in a down-up manner through skip connections, extracting shallow features from the first few layers lacks sufficient contextual information that will be reused in subsequent layers, thus limiting the reconstruction capability of the network. At the same time, skip connections make the neural network deeper, resulting in greatly increased network parameters. Such a large-capacity network occupies a large amount of storage resources and has the problem of over-fitting. To solve these problems give the network a better generalization ability. This work proposes DLRRN with a recursive structure, in which LRL features are corrected by HRL with more contextual information in a top-down flow of information, while LRL information enriches HRL features in a down-top manner. In particular, the recursive structure in the DLRRN (shown in Figure 1b) plays a crucial role in implementing the feedback process.

### 2.2. Feedback Mechanism

The feedback network divides the prediction process of non-linear mapping inputs to the target space into multiple steps, so that the model has a self-correcting ability. In recent years, many network architectures have applied feedback mechanisms to various visual tasks [23,24,25].

Some researchers have made attempts to introduce feedback mechanisms into SR tasks. The DBPN proposed by Haris et al. [23] realizes iterative error feedback through up- and down-projection units. The feedback block (FB) designed in [11] directly iterates convolution and deconvolution to realize (down-) up-sampling, and feedback is realized through the output of FB. To make the feedback mechanism suitable for image SR, this paper carefully designed a CLFFB as the basic module in DLRRN, instead of simple and repeated up- and down-sampling as in [11]. The information in our CLFFB is efficiently inter-corrected between HRL and LRL via cross-layer connections. The experimental results also demonstrate the excellent reconstruction performance of our well-designed CLFFB.

### 2.3. Attention Mechanism

An attention module can model remote dependency and has been widely used in many tasks [11,15,26]. The study of [15] first proposed a self-attention mechanism to describe the global dependencies of inputs and applied it to machine translation. The work [27] introduced self-attention mechanisms to learn better image generators. Subsequently, different attention modules are widely used in computer vision tasks.

The attention module models the features with learning weights to update the features. For example, SENet [28] generates feature vectors in the channel direction through a global pooling operation, then learns the correlation among the channels through feature vectors, highlighting the channel maps with a large amount of information and suppressing unimportant channel features according to different channel weights. CBAM [14] focuses on salient regions by extending the SE module to the spatial dimension. More and more attention mechanisms are used in SR tasks, and SFTGAN [29] adopts a spatial feature transformation layer to make the generated SR images have more realistic and visually pleasing textures. The study of [30] explored the potential of a reference-based super-resolution method on remote sensing images, utilizing rich texture information from HR reference images to reconstruct the details in LR images. The study of [31] learned the predicted convolution kernels and channel modulation coefficients obtained from unsupervised degenerate representations to handle various quantization models. In order to capture rich context and produce visually satisfactory SR images, this paper introduced a self-attention mechanism to SR and crafted SAFEB to better represent features with intra-class compactness.

## 3. Methods

This section introduces the details of our network architecture. Section 3.1 briefly introduces the overall network architecture. Section 3.2 is the basic block (DLRRB) of DLRRN, which is composed of dense CLFFB to handle information flow. Section 3.3 introduces CLFFB, as the core part of our network, which can enhance information by effectively handling cross-layer information flow. SAFEB is introduced in Section 3.4. Because the self-attention mechanism models the spatial position, it is helpful to calculate the loss function of MS-SSIM, thus achieving a better visual effect. Section 3.5 provides a detailed description of the loss function of our network, and this study introduces MS-SSIM [16] to enable the network to produce results that are more consistent with human vision. Finally, the implementation details of our network are shown in Section 3.6.

### 3.1. Network Structure

**Figure 2:**
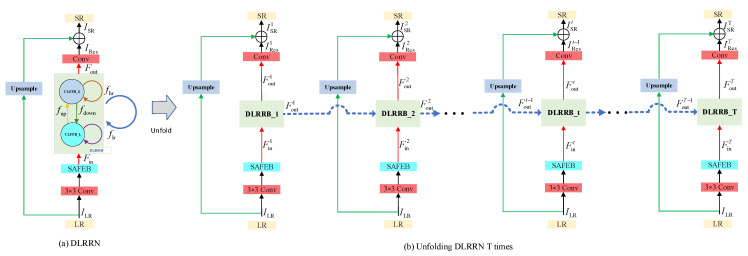
The recurrent structure of the DLRRN is defined as shown in (**a**), and (**b**) is the unfolded DLRRN. Blue arrows represent feedback information flow and green arrows represent global residual skip connections.

Unlike models that work at a single spatial resolution, DLRRN enables pieces of information in LR and HR spaces to be guided to each other. The overall structure of our DLRRN is shown in Figure 2. Specifically, in Figure 2a, CLFFB_L and CLFFB_S represent the LRL information space and HRL information space, respectively. The four colored arrows represent the transfer function between LRL and HRL. There are purple ($f_{lr}$), brown ($f_{hr}$) and yellow $f_{up}\;$arrows exits in conventional RNN, which provide information flow from LRL to LRL, HRL to HRL, and LRL to HRL, respectively. For LRL information to access HRL information with more context information, this paper adds a green arrow ($f_{down}$) to realize the feedback of HRL information.

The DLRRN can be unfolded to ordered T iterations in time, as shown in Figure 2b. In order to make the DLRRN have an early reconstruction ability and carry output information in the feedback information, we established a loss function between each iteration result and HR. The residual branch in each iteration t consists of three parts: shallow feature extraction part (Conv+SAFEB), dual-level recurrent residual block (DLRRB) and dimension reduction block. Each DLRRB is weight-shared in time, while the up-sampled images in each iteration t use global residual skip connections to bypass the residual branch. Therefore, the purpose of the residual branch in each iteration t is to restore the high-resolution residual image $I_{Res}^{t}$ after inputting the low-resolution image $I_{LR}$. In this paper, we used $Conv\left(s,m\right)$ and $Deconv\left(s,m\right)$ to denote the regular convolution and deconvolution layers, respectively, where s and m denote the size and number of filters, respectively. We use $d\;Conv\left(3,4m\right)$ and SAFEB to extract shallow features. In subsequent experiments, we set m to 64 (m=64) by default. We provided the LR image input $I_{LR}$ for LR feature extraction part, and obtained the shallow feature $F_{in}^{t}$ containing LR image information:

(1) $$F_{in}^{t}=SAFEB\left(Conv\left(I_{LR}\right)\right)$$

where $F_{in}^{t}$ is the input of the shallow information of the t-th DLRRB.

The DLRRB of the t-th iteration receives the hidden information $F_{out}^{t-1}$ of the previous iteration and the shallow feature $F_{in}^{t}$, $F_{out}^{t}$ represents the output of DLRRB in the t-th iteration. The mathematical formula of DLRRB is:

(2) $$F_{out}^{t}=H_{DLRRN}\left(F_{out}^{t-1},F_{in}^{t}\right)$$

where $H_{DLRRN}\left(\cdot \right)$ refers to DLRRB operation.

The DLRRB output feature $F_{out}^{t}$ generates a residual image $I_{Res}^{t}$ through a dimension reduction block (DRB). The mathematical formula is:

(3) $$I_{Res}^{t}=Conv\left(F_{out}^{t}\right)$$

where Conv represents the dimension reduction operation.

The output SR image of the *t*-th iteration can be expressed as:

(4) $$I_{SR}^{t}=I_{Res}^{t}+H_{UP}\left(I_{LR}\right)$$

where $H_{UP}$ represents the up-sampling function; therefore, we can choose any up-sampling operation. Here we use bilinear up-sampling operation. After T iterations, we can obtain a total of T SR images $\left(I_{SR}^{1},I_{SR}^{2},\ldots ,I_{SR}^{T}\right)$; we chose $I_{SR}^{T}$ as the final output of our network.

### 3.2. Dual-Level Recurrent Residual Block

**Figure 3:**
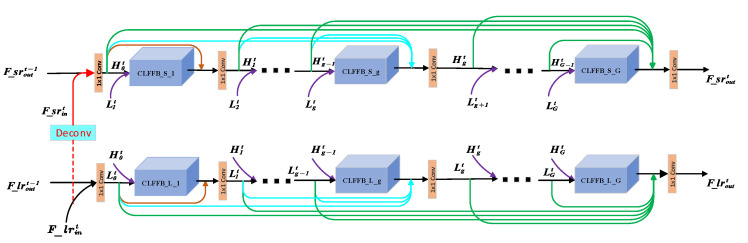
The internal structure of the DLRRB.

The structure of the DLRRB is shown in Figure 3. The DLRRB of the *t*-th iteration receives hidden information $F\_lr_{out}^{t-1}\left(F\_sr_{out}^{t-1}\right)$ to correct the low-level representation $F\_lr_{in}^{t-1}\left(F\_sr_{in}^{t-1}\right)$, and then outputs the high-level representation $F\_lr_{out}^{t}\left(F\_sr_{out}^{t}\right)$ with richer features to the t + 1 iteration and DRB. The DLRRB is composed of G group dense CLFFB, and each CLFFB can make HRL features and LRL features interact to generate final SR images with rich details.

As can be seen from Figure 3, DLRRB contains two branches, one is the SR branch that generates an HRL feature map with rich details through fine LRL feature maps, and the other is the LR branch, which refines LRL feature maps through detailed HRL feature maps. The two branches guide each other and gradually achieve our final image $I_{SR}^{T}$ in rich detail.

At the beginning of the t-th DLRRB, the LR branch receives the input information $F\_lr_{in}^{t}$ and output information $F\_lr_{out}^{t-1}$ of the previous layer, and then concatenates and compresses them by $Conv\left(1,m\right)$ to generate a rough input feature map $L_{0}^{t}$:

(5) $$L_{0}^{t}=C_{0}^{l}\left(\left[F\_lr_{in}^{t},F\_lr_{out}^{t-1}\right]\right)$$

Similarly:

(6) $$H_{0}^{t}=C_{0}^{h}\left(\left[F\_sr_{in}^{t},F\_sr_{out}^{t-1}\right]\right)$$

where $F\_lr_{in}^{t}$ is $F_{in}^{t}$ in Figure 2, $F\_sr_{in}^{t}=Deconv\left(F\_lr_{in}^{t}\right)$, Deconv is $Deconv\left(s,m\right)$, $\left[F\_lr_{in}^{t},F\_lr_{out}^{t-1}\right]$ refers to the concatenations of $F\_lr_{in}^{t}$ and $F\_lr_{out}^{t-1}$, and $C_{0}^{l\left(h\right)}$ represents the initial dimensionality reduction operation using $Conv\left(1,m\right)$ in LR(SR) branch.

$L_{g}^{t}$ and $H_{g}^{t}$ represent the LRL and HRL feature map output of the g-th CLFFB of DLRRB in the t-th iteration, respectively. $L_{g}^{t}$ can be expressed as:

(7) $$L_{g}^{t}=C_{g}^{l}\left(\left[L_{0}^{t},L_{1}^{t},\ldots ,L_{g-1}^{t},f\_lr_{g}^{t}\right]\right)$$

where $C_{g}^{l}$ indicates that $Conv\left(1,m\right)$ is used for dimension reduction in the g-th feature fusion group in LR branch, and$\;f\_lr_{g}^{t}$ indicates the feature maps the output of the g-th CLFFB in the t-th iteration (see Figure 3).

Similarly:

(8) $$H_{g}^{t}=C_{g}^{h}\left(\left[H_{0}^{t},H_{1}^{t},\ldots ,H_{g-1}^{t},f\_sr_{g}^{t}\right]\right)$$

To use useful information from each group and to correct the input features $F_{in}^{t+1}$ for the next iteration, we fuse the feature maps of each group (green arrows in Figure 3). the output of DLRRB as follows:

For the LRL:

(9) $$F\_lr_{out}^{t}=C_{FF}^{l}\left(\left[L_{0}^{t},L_{1}^{t},\ldots ,L_{G}^{t}\right]\right)$$

For the HRL:

(10) $$F\_sr_{out}^{t}=C_{FF}^{h}\left(\left[H_{0}^{t},H_{1}^{t},\ldots ,H_{G}^{t}\right]\right)$$

where $F\_sr_{out}^{t}$ is $F_{out}^{t}$ in Figure 2. $C_{FF}^{l\left(h\right)}\left(\cdot \right)$ represents the feature fusion of the last layer of the t-th DLRRB in the LR(SR) branch, which is expressed as $Conv\left(1,m\right)$ function.

It is worth mentioning that in the first DLRRB in the DLRRN, we initialize as follows.

For LR branch:

(11) $$F\_lr_{in}^{1}=F_{in}^{1},F\_lr_{out}^{0}=F\_lr_{in}^{1}$$

For SR branch:

(12) $$F\_sr_{in}^{1}=Deconv(F\_lr_{in}^{1}),F\_sr_{out}^{0}=F\_sr_{in}^{1}$$

### 3.3. Cross-Level Feature Fusion Block

**Figure 4:**
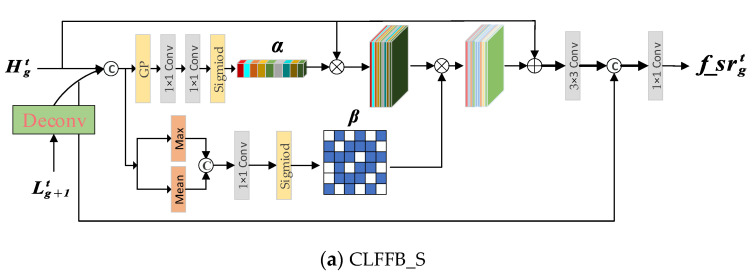

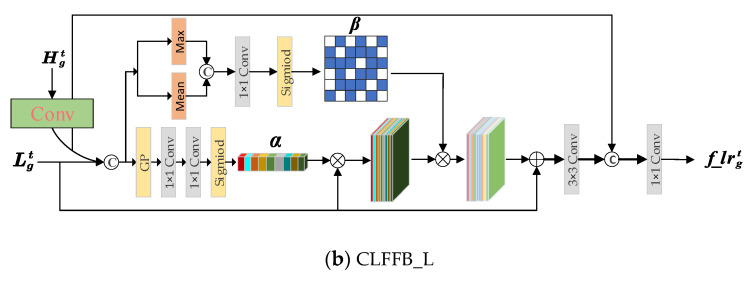
The proposed CLFFB, (**a**) is the SR branch represented as CLFFB_S, (**b**) is the LR branch denoted as CLFFB_L.

Different from the study of [12,23], which directly fuses low-level and high-level features, we use the cross-layer feature gate mechanism to guide selectively enhanced spatial details. Therefore, we propose an effective CLFFB (as shown in Figure 4) to process the information flow in the network.

Specifically, the input of the CLFFB (the following takes CLFFB_L as an example) includes two parts. One part is that the feature map $H_{g}^{t}$ from the SR branch is resized to the same size as $L_{g}^{t}$ by a convolution operation, and the previous output $L_{g}^{t}$ from the LR branch jointly generates the cross-level feature map $l_{g}^{t}$:

(13) $$l_{g}^{t}=\left[{\left(H_{g}^{t}\right)}^{↓},L_{g}^{t}\right]$$

where ${\left(H_{g}^{t}\right)}^{↓}$ indicates the downsampling operation of $H_{g}^{t}$.

We feed the generated cross-layer feature map $l_{g}^{t}$ into two branches to refine the LRL features. One branch is to generate the weight vector α to reweight the features in the channel direction:

(14) $$\alpha =Sigmoid\left(conv\left(conv\left(avgpool\left(l_{g}^{t}\right)\right)\right)\right)$$

where $avgpool\left(\cdot \right)\;$represents the global average pooling function, conv represents $conv\left(1,m\right)$, and Sigmoid refers to the Sigmoid activation function.

The other branch is used to generate an attention map $\beta \in R^{H\times W}$:

(15) $$\beta =Sigmoid(conv\left(conv\left(\left[Mean\left(l_{g}^{t}\right),Max\left(l_{g}^{t}\right)\right]\right)\right)$$

where Mean, Max is the average and maximum pooling function along the channel axis, and conv is the $conv\left(1,1\right).$

The generated weight vector α, attention map β and feature map $L_{g}^{t}$ are summed and multiplied element-wise to obtain a fine feature map, and cascaded with the cross-level feature map ${\left(H_{g}^{t}\right)}^{↓}$, and then the output $f\_lr_{g}^{t}$ of CLFFB is obtained through a convolution layer.

(16) $$f\_lr_{g}^{t}=conv\left(\left[Conv\left(L_{g}^{t}⨀\left(1+\alpha ⨀\beta \right)\right),{\left(H_{g}^{t}\right)}^{↓}\right]\right)$$

where ⨀ is the element-wise product, ${\left(\cdot \right)}^{↓}$ is the downsampling operation, Conv is $Conv\left(3,m\right)$, and conv is $conv\left(1,m\right)$.

### 3.4. Self-Attention Feature Extraction Block

**Figure 5:**
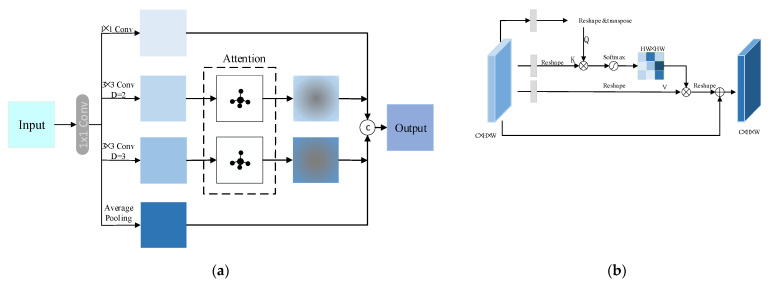
(**a**) Our proposed SAFEB and (**b**) the structure of the self-attention mechanism.

The scale of objects in LR images is varied, and single-scale features cannot capture multi-scale contextual information of different objects. Since the non-salient regions are relatively dispersed, the direct aggregation of multi-scale features may weaken the representation ability of important regions. We separately placed self-attention [15] (the structure is shown in Figure 5b) on different scales of features in order to focus more attention on visually important areas; therefore, we have constructed SAFEB, as shown in Figure 5a.

We first load the input low-level feature maps in parallel to the dilated convolution layers with different dilation rates to extract rich features, then add self-attention mechanism modules [15] (as shown in Figure 5b) to each branch. The input and output of the self-attention block are denoted as $F\_att_{in}=R^{m\times H\times W}$ and $F\_att_{out}=R^{m\times H\times W}$, respectively. The attention map A can be obtained by:

(17) $$A=softmax\left(R_{1}{\left(Conv\left(F_{{att}_{in}}\right)\right)}^{T}\times R_{1}\left(Conv\left(F\_att_{in}\right)\right)\right))$$

where $softmax\left(\cdot \right)$ is the softmax function, $R_{1}\left(\cdot \right)$ indicates that the reshape input feature is $R^{C\times N},N=H\times W$.

Next, we combine the attention features maps A with $F\_att_{in}$ to generate enhanced attention feature maps, then add the input feature maps $F\_att_{in}$ to obtain the final output $F\_att_{out}$ as follows:

(18) $$F\_att_{out}=F\_att_{in}+R_{2}\left(R_{1}\left(Conv\left(F\_att_{in}\right)\right)\times A^{T}\right)$$

where $R_{2}\left(\cdot \right)$ refers to reshape input features to $R^{C\times H\times W}$.

In particular, we do not apply the self-attention module to the global average pooling branch and 1×1 convolution branch because these two branches are designed to use the minimum and maximum receptive fields to keep the intrinsic properties of the input.

### 3.5. Loss Function

In deep neural networks, the loss function is the essential part, which determines the direction of our network optimization. We use the L1 loss function and MS-SSIM [16] loss function to optimize our network. The results show that our network can produce a better visual effect without reducing objective performance metrics (PSNR, SSIM), and achieve the balance between perception and objective evaluation metrics.

In the evaluation index of image quality, PSNR and SSIM [32] is generally used as the evaluation index for images generated by L1 and L2 loss function optimization networks, but L1 and L2 have one thing in common: they are based on per-pixel comparison of differences, without considering human visual perception, and without considering human aesthetics, so a high PSNR value does not mean a good visual quality of an image. In [16], the structural similarity loss function (SSIM) and multi-scale structural similarity loss function (MS-SSIM) are designed to restore images with better vision. The SSIM loss function considers luminance, contrast and structure, which takes human visual perception into account. Generally speaking, the results obtained by SSIM are better than those obtained by L1 and L2 in visual.

SSIM for a certain pixel p is defined as:

(19) $$SSIM\left(p\right)=\frac{2\mu_{x}\mu_{y}+C1}{\mu_{x}^{2}+\mu_{y}^{2}+C1}\cdot \;\frac{2\delta_{xy}+C2}{\delta_{x}^{2}+\delta_{y\;}^{2}+C2}=l\left(p\right)\cdot cs\left(p\right)$$

where *x*,y represents the processed image and the real image, $\mu_{x\left(y\right)}$ is the mean value of X(Y), $\delta_{x\left(y\right)}^{2}$ represents the variance of X (Y), $\delta_{xy}$ is the covariance of X and Y, C1 and C2 are constants, and its calculation formula is $C1={\left(k_{1}L\right)}^{2}$ and $C2={\left(k_{2}L\right)}^{2}$, L is the gray value range of the image ([0, 255] for color images and [0, 1] for gray images). $k_{1}$ and $k_{2}$ are two constants, and the default values are 0.01 and 0.03. It should not be overlooked that the mean and standard deviation are calculated by the Gaussian filter.

We can learn from [16] that it is crucial to choose the size of Gaussian kernel to calculate the mean and variance of images in SSIM. If it is chosen to be small, the calculated SSIM loss cannot keep the local structure of the image well, and artifacts will appear. If the selection is large, the network will produce noise at the edge of the image. In order to avoid time-consuming adjustments of Gaussian kernel size, [16] proposed a version of multi-scale SSIM, and MS-SSIM is defined as:

(20) $$MS-SSIM\left(p\right)=l_{M}^{\alpha }\left(p\right)\cdot \prod_{j=1}^{M}cs_{j}^{\beta_{j}}\left(p\right)$$

where $l_{M}$ and $cs_{j}$ represent Equation (19) at the scale of M and j, respectively. For convenience, $\alpha =\beta_{j}=\left\{1\right\},j=\left\{1,\;.\;.\;.\;,\;M\right\}$.

Therefore, the loss function of MS-SSIM is:

(21) $$L^{MS-SSIM}\left(p\right)=1-MS-SSIM\left(\tilde{p}\right)$$

where $\tilde{p}$ is the center pixel of input image patch P.

We combine *L*1 with MS-SSIM as the loss function of our network, which is defined as $L_{DLRRN}$:

(22) $$\;\;\;\;\;\;L_{DLRRN}\left(\omega \right)=L_{1}+\omega L^{MS-SSIM}\;=\frac{1}{T}\overset{T}{\underset{t=1}{\sum }}(\|I_{HR}^{t}-I_{SR}^{t}{\|}_{1}+\theta L^{MS-SSIM}\left(I_{HR}^{t},\;I_{SR}^{t}\right))$$

where the θ indicates the trade-off factor, ω denotes the parameters of the DLRRN, and $I_{HR}^{0}=I_{HR}^{1}=\ldots =I_{HR}^{T}$ represents the SR image reconstructed by the *t*-th iteration.

### 3.6. Network Details

The activation function after the convolution layer and deconvolution layer is PRelu [32]. As with [12], we set different k in the $\left(De\right)Conv\left(k,m\right)$ according to different scaling factors to achieve (up-)downsampling of the feature map, as shown in Table 1. We can obtain a total of T SR images $\left(I_{HR}^{0},I_{HR}^{1},\cdots ,I_{SR}^{T}\right)$, and we chose $I_{SR}^{T}$ as the final output of our network. Our network can handle both grey and color images, the output channel of the last convolution layer can be 1 or 3, accordingly.

## 4. Experimental Section

In this chapter, we describe the experimental process and analyze results in detail. The public datasets, evaluation metrics, degradation model, training settings and experimental conditions are described in Section 4.1. Section 4.2 is the experimental analysis. Firstly, we study the influence of iteration times T and the number G of CLFFB_L and CLFFB_S in CLFFB on the reconstruction performance. Secondly, we analyze the loss function. Finally, we explore the influence of SAFEB on the experimental results. Section 4.3 describes the algorithm comparison and visualization results. We first analyze the network parameters and the complexity. The training results of the network’s training models (BI × 2, BI × 3, BI × 4, BD × 3, DN × 3) were then compared with those of other algorithms.

### 4.1. Implementation Details

We use DIV2K [33] as the training dataset of the network, which contains 800 training images and 100 validation images. To make our trained model more robust, there are two ways to augment the data, as described in [14]: (1) scaling—reducing the scale [0.8, 0.7, 0.6, 0.5]; (2) rotation and flip—horizontally flipping and rotating 90 degrees to expand the training data. We evaluated SR results for five standard benchmark datasets under PSNR and SSIM [32] indicators: Set5 [25], Set14 [34], BSD100 [35], Urban100 [36] and Manga109 [37]. As in previous work, our experimental results were quantitatively evaluated in the luminance (Y) channel.

To ensure a fair comparison with previous work, we used the process of HR obtaining LR by bicubic downsampling as the standard degradation (denoted as BI). To verify the generalization ability of our network to deal with multiple degradation models, we further experimented with two additional degradation models BD and DN [22]. BD is defined as firstly blurring HR image with Gaussian kernel with size 7 × 7 and standard deviation of 1.6, and then performing downsampling operation. DN is defined as the process of first adding Gaussian noise with a noise level of 30 to the HR and then obtaining the LR by standard bicubic downsampling. BI/BD/DN × n means that HR is degraded by BI/BD/DN and the downsampling factor is n to obtain LR, and the formed LR-HR image pair is used for network training or testing, as shown in Table 2.

In our training process, we set the input batchsize to 8. In order to make the extracted features contain more LR image context information, similar to the study of [12], we set different patchsizes for different scaling factors (Table 3 lists the input patchsize settings). Using the method in [24] to initialize the network parameters, we used Adam [26] as the optimization function for our network. The initial learning rate of our network was 0.0001, and was halved every 150 epochs; we trained a total of 600 epochs. We used the Pytorch framework to realize our network and train it at TITAN RTX.

### 4.2. Experimental Analysis

#### 4.2.1. Study of T and G

**Figure 6:**
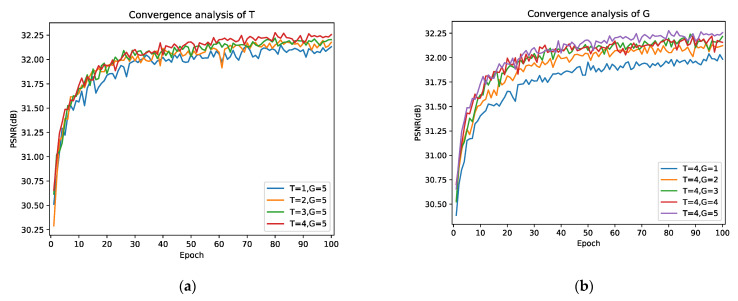
Convergence analysis of T and G on Set5 with BI × 4. (**a**) is the convergence curve with respect to T, (**b**) is the convergence curve with respect to G.

**Figure 7:**
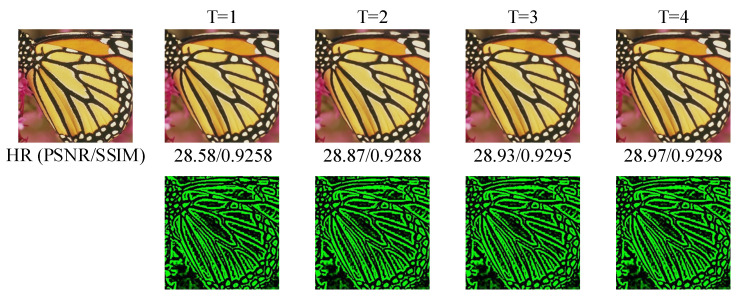
In the test of the best model of BI × 4, the first group indicates that the reconstruction performance improves with the increase in T. The second group is its corresponding residual map $I_{Res}^{T}$.

In this subsection, we will discuss the effect of the iterations times (denoted as T) and the number of groups (denoted as G) of CLFFB_L and CLFFB_S in the DLRRB on the reconstruction results. We first set G = 5 to analyze the effect of T on the reconstruction results, and the experimental results are shown in Figure 6a. It highlights the fact that the reconstruction quality increases with T. In general, the reconstruction performance of the network is outstanding; therefore, CLFFB is effective for the SR task. In addition, we visualized T on the BI × 4 model (as shown in Figure 7, the first group is the reconstructed RGB image, and the second group is its corresponding residual image ($I_{Res}^{T}$). Then, T = 4 is allowed to study the influence of G on network reconstruction, and its convergence curve is shown in Figure 6b. We can find that the larger the G value, the better the reconstruction performance, indicating that the deep network has a strong representation capability. Overall, choosing a larger T or G is helpful to obtain better results. In the following discussion, we use DLRRN (T = 4, G = 5) for analysis. It is worth mentioning that we consider both network performance and network parameters, so we assume T = 4 and G = 5.

#### 4.2.2. Analysis of Loss Function

**Figure 8:**
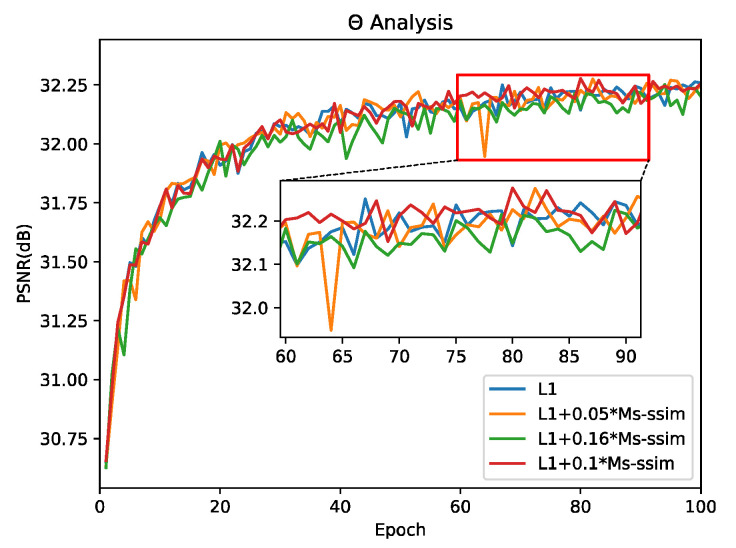
Effect of the hyper-parameter θ in Equation (22) on the performance of $L^{MS-SSIM}$ (testing on Set5 for BI × 4).

**Figure 9:**
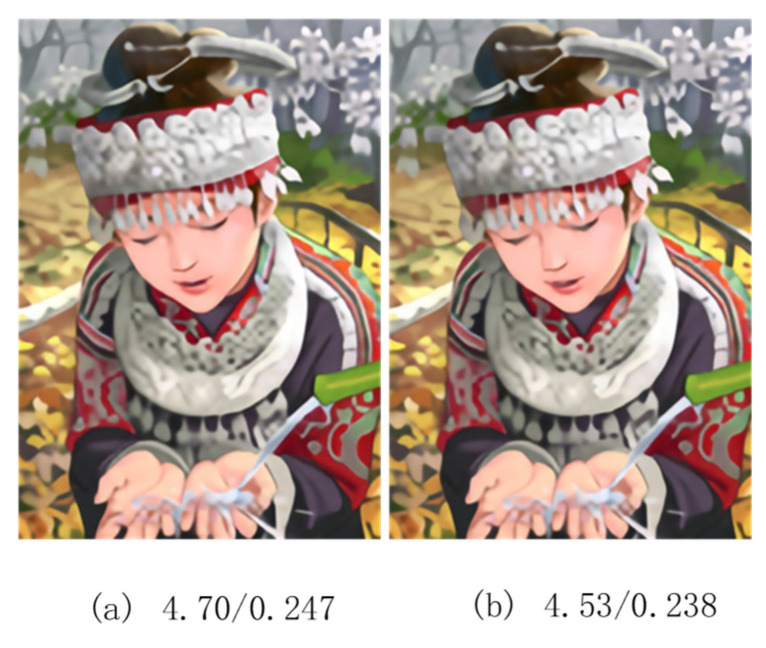
(**a**) SR images generated only by L1 loss function training. (**b**) SR image generated by L1 and MS-SSIM joint loss function training.

We uses Equation (22) $L_{DLRRN}$ as the loss function of our optimized network. We first explored the influence of hyperparameter θ on the training of our network. We used the dichotomy method to explore the value range of θ as shown in Figure 8, and the experimental results showed that when θ=0.1, the training results could reach the relative optimal solution (i.e., the PSNR value was relatively maximum). At the same time, our network was compared with the L1-trained network alone, and the results showed that the results of the $L_{DLRRN}$ training were slightly higher than the results of the L1 loss training in the objective evaluation metrics (32.28 vs. 32.26 from Figure 8). Additionally, we proved that, when the results of the $L_{DLRRN}$ and the L1 training were the same as the PSNR, due to MS-SSIM, our network produced superior visual effects, as shown in Figure 9 (the visual evaluation metrics, PI [38] and LPIPS [39], are shown at the bottom of the figure) and in Table 4.

#### 4.2.3. Ablation Analysis of SAFEB

Regarding the ablation analysis of SAFEB, we could use $Conv\left(1,m\right)$ a convolution layer to place SAFEB as our baseline. As shown in Table 5, we could obtain the following results through experiments: within 100 epochs, when SAFEB acted on the network alone, the reconstruction performance was slightly increased. Our network performance was improved by 0.04 dB (32.38 vs. 32.42) when we experimented with 200 epochs, which shows that it is effective for SAFEB.

Although SAFEB acting alone on the network did not significantly improve performance, our experiments showed that it could improve the visual effects. As shown in Table 4, we used the BI × 4 model to test on Set5 under PSNR = 32.40, and we used PI [38] and LPIPS [39] as evaluation metrics of visual quality, which showed the effectiveness of SAFEB and MS-SSIM in improving visual effect.

### 4.3. Comparison with Previous Work

#### 4.3.1. Network Parameters and Complexity

**Figure 10:**
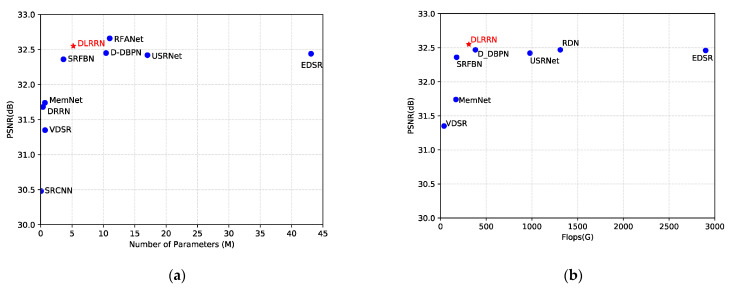
(**a**) PSNR and parameters. The results are the BI × 4 evaluated on Set5. Red points indicate our proposed network and achieved relatively optimal performance. (**b**) PSNR and Flops. Flops are computed on 720p HR images.

We compared DLRRN with ten deep-learning-based SR methods: SRCNN [10], VDSR [6], DRRN [8], MemNet [40], EDSR [19], DBPN-S [23], D-DBPN [24], SRFBN [12], USRNet [41] and RFANet [42]. The comparison results of network parameters and reconstruction effect (PSNR) are shown in Figure 10. We can see from Figure 10a that the network parameters and reconstruction performance of our method are relatively optimal. Our network requires only 35% and 8% of the parameters in the D-DBPN and EDSR, while achieving better reconstruction results. Although RFANet has a slightly higher performance than our network, its number of parameters is twice that of our network. Overall, compared with other latest methods, our network is lighter and more efficient.

We compared DLRRN’s Flops with other algorithms, and the comparison results are shown in the Figure 10b. It can be seen from the figure that, compared with SRFBN, the Flops of the algorithm in this paper increases by 75%, and its performance is improved by 0.19 dB. Compared with USRNet, the Flops of this algorithm is reduced by 68%, and it can achieve comparable performance. Overall, Flops also reflects the effectiveness of our algorithm to some extent. Since the algorithm in this paper works in the LR and HR spaces and adopts a dense structure, it leads to more computational complexity of the network. Next, we will try to drastically reduce the complexity of the network without affecting the reconstruction effect.

#### 4.3.2. Results of Evaluation on BI Model

We compare DLRRN with the ten latest image SR methods: SRCNN [10], VDSR [6], DRRN [8], SRDenseNet [20], MemNet [40], EDSR [19], D-DBPN [23], SRFBN [12], USRNet [41] and RFANet [42]. The results of quantitative evaluation are shown in Table 6. Compared with our method, EDSR uses more filters (256 v.s. 64), while D-DBPN, USRNet and DRN use more training images (DIV2K + Flickr2K v.s. DIV2K). Compared with them, however, our DLRRN can obtain competitive results.

**Figure 11:**
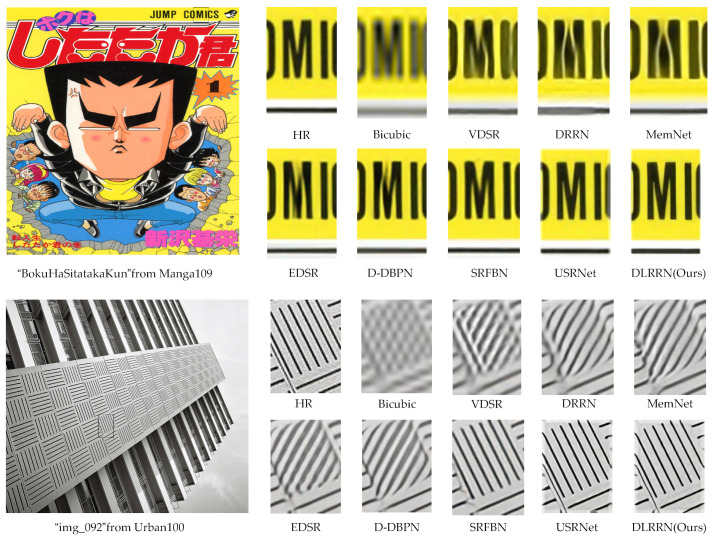
Comparison of the visual effect of the method in this paper with other methods on BI × 4.

We show the SR visualization results of BI × 4 in Figure 11. The proposed DLRRN can produce more convincing results (as the RFANet code is not open source, we do not have access to its visuals). We can see from SR visualization results of the “BokuHaSitatakaKun” image in Manga109 that “M” letters reconstructed by DRRN and MemNet are separated, the VDSR, EDSR and D-DBPN cannot restore the clear texture of the image, the image generated by SRFBN is fuzzy, and the image edge restored by USRNet has many artifacts. The proposed DLRRN produces clear images, even smoother than the label. In addition, we also visualized “img 092” in Urban100, the texture directions of SR images reconstructed by other comparison methods except SRFBN and USRNet are all wrong. However, our proposed DLRRN allows HRL information and LRL information to be mutually corrected in the iterative process and optimizes our network by using L1 and MS-SSIM loss functions, so the obtained SR image is smoother than the ground truth and more in line with people’s vision.

#### 4.3.3. Results of Evaluation on BD and DN Models

To verify the generalization ability of our network model, the proposed DLRRN is also trained in BD and DN degradation models and DLRRN with SRCNN [10], VDSR [6], IRCNN_G [43], IRCNN_C [43], SRMD(NF) [44], RDN [22], SRFBN [12] and RFANet are compared [42]. The results of the quantitative evaluation with the latest algorithm are shown in Table 7. We find that our algorithm performs well on most datasets.

**Figure 12:**
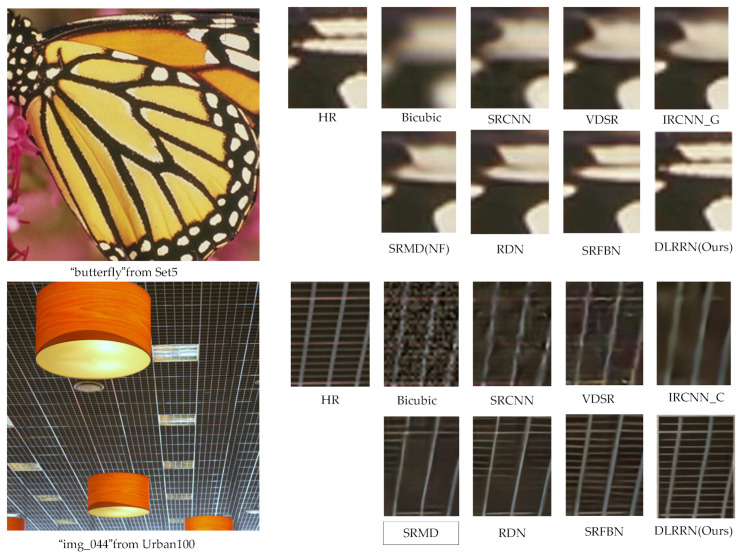
The visualization results of BD × 3 and DN × 3; the first group and the second group represent the results of BD × 3 and DN × 3, respectively.

We show two groups of SR visual results tested on the BD and DN models in Figure 12. From the visualization results, we can see that our network can reduce distortion and recover SR images with more details. From the overall experimental results, it is concluded that our network handles BD and DN degradation more robustly and effectively.

## 5. Conclusions and Discussion

In this paper, we realize image super-resolution reconstruction by adding an extra level in the super-resolution network based on feedforward structure, called super-resolution via dual-level recurrent residual network (DLRRN), which makes the pieces of HRL information and LRL information guide each other through the iterative process, so as to achieve the better reconstruction of SR images. The proposed CLFFB plays an important role in the iterative process, which is used to effectively fuse the cross-level information flow and features enhancement. We use the combination of L1 and $L^{MS-SSIM}$ loss function to make an attempt to trade-off objective performance measures and visual effects. In conclusion, our comprehensive experimental results show that the proposed DLRRN has a good effect on the objective evaluation index and visual effects.

**Figure 13:**
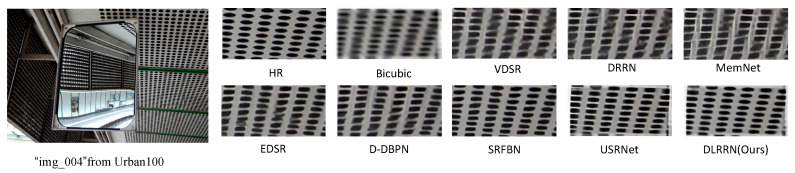
The visualization results of BI × 4, the visual effect restored in the middle area of the image is very clear, but it is a little blurred at the edge of the image.

However, the method proposed in this paper has the limitation of a high complexity compared to a pure feed-forward network (i.e., The high-level feature learning stage only works in the LR space.) due to the dense structure and working in both HR and LR spaces. Our experimental results show that (as shown Figure 13) the SR image generated by our network can produce a good visual effect for the middle area of the image, but the restoration effect for the edge of the image is not ideal. We find that Equation (13) emphasizes that the calculation of the standard deviation in SSIM(p) needs the support of pixel neighborhood, and SSIM(p), and its derivatives cannot be calculated in some boundary regions of p. In conclusion, our comprehensive experimental results show that the proposed DLRRN has a good effect on objective evaluation index and visual effect. Next, our work will continue to explore the situation of satisfying visual effects and recovering better edge information.

In future studies, we will explore the lightweight aspects of the SR network and try to introduce a non-parametric attention mechanism or dynamic convolution layer to enhance information extraction in the high-level information learning stage of the network. We will improve the reconstruction block of the network and design a more efficient reconstruction part instead of simply using transposed convolution or sub-pixel convolution. At the same time, in the future work, we will apply this work to video SR or introduce it into the real world for real-time broadcasting.

## Figures and Tables

**Table 1:** Different scaling factors correspond to different kernel_size, padding, stride.

Scale	Kernel_Size	Padding	Stride
2	6	2	2
3	7	2	3
4	8	2	4

**Table 2:** Degradation model experiments conducted in this paper.

Degeneration	Definition
BI×2	Under BI degradation, the scaling factor is 2.
BI×3	Under BI degradation, the scaling factor is 3.
BI×4	Under BI degradation, the scaling factor is 4.
DN×3	Under DN degradation, the scaling factor is 3.
BD×3	Under BD degradation, the scaling factor is 3.

**Table 3:** Different scaling factors correspond to different kernel_size, padding, stride.

Scale	×2	×3	×4
Input patchsize	60× 60	50× 50	40× 40

**Table 4:** Ablation analysis of SAFEB.

	DLRRN	$\mathrm{DLRRN}\text{-}L^{MS-SSIM}$	DLRRN-SAFEB
Set5 (PI/LPIPS)	5.944/0.1730	6.054/0.1745	6.123/0.1745

**Table 5:** Ablation analysis of SAFEB.

	a	b
Base	√	
Base + SAFEB		√
PSNR on Set5 (BI×4)	32.26	32.28

**Table 6:** Quantitative evaluation of comparative algorithms in BI degradation models. Red indicates the best SR reconstruction performance, and blue is the second best.

Scale	Method	Set5 PSNR/SSIM	Set14 PSNR/SSIM	BSD100 PSNR/SSIM	Urban100 PSNR/SSIM	Manga109 PSNR/SSIM
2	Bicubic	33.66/0.9299	30.24/0.8688	29.56/0.8431	26.88/0.8403	30.30/0.9339
	SRCNN [10]	36.66/0.9542	32.45/0.9067	31.36/0.8879	29.50/0.8946	35.60/0.9663
	VDSR [6]	37.53/0.9590	33.05/0.9130	31.90/0.8960	30.77/0.9140	37.22/0.9750
	DRRN [8]	37.74/0.9591	33.23/0.9136	32.05/0.8973	32.23/0.9188	37.60/0.9736
	MemNet [40]	37.78/0.9597	33.28/0.9142	32.08/0.8978	31.31/0.9195	37.72/0.9740
	EDSR [19]	38.11/0.9602	33.92/0.9195	32.32/0.9013	32.93/0.9351	39.10/0.9773
	D-DBPN [23]	38.09/0.9600	33.85/0.9190	32.27/0.9000	32.55/0.9324	38.89/0.9775
	SRFBN [12]	38.02/0.9601	33.74/0.9190	32.21/0.9004	32.53/0.9320	38.99/0.9771
	USRNet [41]	37.71/0.9592	33.49/0.9156	32.10/0.8981	31.79/0.9255	38.37/0.9760
	RFANet [42]	38.26/0.9615	34.16/0.9220	32.41/0.9026	33.33/0.9389	39.44/0.9783
	DLRRN (ours)	38.19/0.9612	34.05/0.9219	32.33/0.9012	33.02/0.9357	39.24/0.9783
3	Bicubic	30.39/0.8682	27.55/0.7742	27.21/0.7385	24.46/0.7349	26.95/0.8556
	SRCNN [10]	32.75/0.9090	29.30/0.8215	28.41/0.7863	26.24/0.7989	30.48/0.9117
	VDSR [6]	33.67/0.9210	29.78/0.8320	28.83/0.7990	27.14/0.8290	32.01/0.9340
	DRRN [8]	34.03/0.9244	29.96/0.8349	28.95/0.8004	27.53/0.8378	32.42/0.9359
	MemNet [40]	34.09/0.9248	30.00/0.8350	28.96/0.8001	27.56/0.8376	32.51/0.9369
	EDSR [19]	34.65/0.9280	30.52/0.8462	29.25/0.8092	28.80/0.8653	34.17/0.9476
	D-DBPN [23]	-/-	-/-	-/-	-/-	-/-
	SRFBN [12]	34.59/0.9283	30.45/0.8450	29.16/0.8071	28.58/0.8628	34.03/0.9462
	USRNet [41]	34.43/0.9279	30.51/0.8446	29.18/0.8076	28.38/0.8575	34.05/0.9466
	RFANet [42]	34.79/0.9300	30.67/0.8487	29.34/0.8115	29.15/0.8720	34.59/0.9506
	DLRRN	34.74/0.9297	30.61/0.8473	29.27/0.8088	29.06/0.8684	34.32/0.9489
4	Bicubic	28.42/0.8104	26.00/0.7027	25.96/0.6675	23.14/0.6577	24.89/0.7866
	SRCNN [10]	30.48/0.8628	27.50/0.7513	26.90/0.7101	24.52/0.7221	27.58/0.8555
	VDSR [6]	31.35/0.8830	28.02/0.7680	27.29/0.7260	25.18/0.7540	28.83/0.8870
	DRRN [8]	31.68/0.8888	28.21/0.7721	27.38/0.7284	25.44/0.7638	29.18/0.8914
	MemNet [40]	31.74/0.8893	28.26/0.7723	27.40/0.7281	25.50/0.7630	29.42/0.8942
	EDSR [19]	32.46/0.8968	28.80/0.7876	27.71/0.7420	26.64/0.8033	31.02/0.9148
	D-DBPN [23]	32.47/0.8980	28.82/0.7860	27.72/0.7400	26.38/0.7946	30.91/0.9137
	SRFBN [12]	32.36/0.8970	28.77/0.7863	27.67/0.7392	26.49/0.7979	30.99/0.9142
	USRNet [41]	32.42/0.8978	28.83/0.7871	27.69/0.7404	26.44/0.7976	31.11/0.9154
	RFANet [42]	32.66/0.9004	28.88/0.7894	27.79/0.7442	26.92/0.8112	31.41/0.9187
	DLRRN (ours)	32.55/0.8994	28.90/0.7887	27.74/0.7408	26.82/0.8057	31.38/0.9176

**Table 7:** Quantitative evaluation results in BD × 3 and DN × 3: Red indicates optimal PSNR/SSIM, and blue is the next best.

Method	Model	Set5 PSNR/SSIM	Set14 PSNR/SSIM	BSD100 PSNR/SSIM	Urban100 PSNR/SSIM	Manga109 PSNR/SSIM
Bicubic	BD	28.34/0.8161	26.12/0.7106	26.02/0.6733	23.20/0.6661	25.03/0.7987
	DN	24.14/0.5445	23.14/0.4828	22.94/0.4461	31.63/0.4701	23.08/0.5448
SRCNN [10]	BD	31.63/0.8888	28.52/0.7924	27.76/0.7526	25.31/0.7612	28.79/0.8851
	DN	27.16/0.7672	25.49/0.6580	25.11/0.6151	23.32/0.6500	25.78/0.7889
VDSR [6]	BD	33.30/0.9159	29.67/0.8269	28.63/0.7903	26.75/0.8145	31.66/0.9260
	DN	27.72/0.7872	25.92/0.6786	25.52/0.6345	23.83/0.6797	26.41/0.8130
IRCNN_G [43]	BD	33.38/0.9182	29.73/0.8292	28.65/0.7922	26.77/0.8154	31.15/0.9245
	DN	24.85/0.7205	23.84/0.6091	23.89/0.5688	21.96/0.6018	23.18/0.7466
IRCNN_C [43]	BD	29.55/0.8246	27.33/0.7135	26.46/0.6572	24.89/0.7172	28.68/0.8574
	DN	26.18/0.7430	24.68/0.6300	24.52/0.5850	22.63/0.6205	24.74/0.7701
SRMD(NF) [44]	BD	34.09/0.9242	30.11/0.8364	28.98/0.8009	27.50/0.8370	32.97/0.9391
	DN	27.74/0.8026	26.13/0.6974	25.64/0.6495	24.28/0.7092	26.72/0.8424
RDN [22]	BD	34.57/0.9280	30.53/0.8447	29.23/0.8079	28.46/0.8581	33.97/0.9465
	DN	28.46/0.8151	26.60/0.7101	25.93/0.6573	24.92/0.7362	28.00/0.8590
SRFBN [12]	BD	34.65/0.9283	30.64/0.8435	29.18/0.8066	28.43/0.8578	34.02/0.9462
	DN	28.52/0.8180	26.58/0.7140	25.94/0.6615	24.96/0.7120	27.98/0.8612
RFANet [42]	BD	34.77/0.9292	30.68/0.8473	29.34/0.8104	28.89/0.8661	34.49/0.9492
	DN	-\-	-\-	-\-	-\-	-\-
DLRRN(ours)	BD	34.80/0.9295	30.68/0.8469	29.32/0.8094	28.95/0.8658	34.57/0.9490
	DN	28.64/0.8210	26.70/0.7147	26.00/0.6630	25.24/0.7485	28.24/0.8650

## Data Availability

Publicly available datasets were analyzed in this study. Our training set DIV2k can be obtained from: https://data.vision.ee.ethz.ch/cvl/DIV2K/ (accessed on 18 October 2021). The URLs of test sets Set5, Set14, BSD100, Urban 100 and Manga109 (Low-Complexity Single-Image Super-Resolution (inria.fr)) are available online at: https://sites.google.com/site/romanzeyde/research-interests (accessed on 18 October 2021), https://www.eecs.berkeley.edu/Research/Projects/CS/vision/bsds/ (accessed on 18 October 2021), https://sites.google.com/site/jbhuang0604/publications/struct_sr (accessed on 18 October 2021) and http://www.manga109.org/en/, respectively (accessed on 18 October 2021).

## Abbreviations

LR (HR)	Low-(high-)resolution
DLRRB	The dual-level recurrent residual block
HRL (LRL)	HR-level (LR-level)
CLFFB_S/CLFFB_L	Cross-level feature fusion block of HRL/(LRL)
SAFEB	The self-attention feature extraction block
CLFFB	Collectively referred to as CLFFB_S and CLFFB_L
DRB	dimension reduction block
BI	The process of obtaining LR image by bicubic downsampling of HR image.
BD	First blurring the HR image with a Gaussian kernel with size 7×7 and standard deviation of 1.6, and then performing a downsampling operation
DN	The process of first adding Gaussian noise with a noise level of 30 to the HR and then obtaining the LR by standard bicubic downsampling
